# In vitro permissivity of bovine cells for wild-type and vaccinal myxoma virus strains

**DOI:** 10.1186/1743-422X-4-94

**Published:** 2007-09-27

**Authors:** Béatrice Pignolet, Jean-Luc Duteyrat, Aude Allemandou, Jacqueline Gelfi, Gilles Foucras, Stéphane Bertagnoli

**Affiliations:** 1 Laboratory « Interactions Hôtes-Virus et Vaccinologie », UMR 1225 INRA-ENVT, Ecole Nationale Vétérinaire de Toulouse, 23 chemin des capelles, Toulouse F-31076, France; 2 laboratory « Résistome des ruminants », UMR 1225 INRA-ENVT, Ecole Nationale Vétérinaire de Toulouse, 23 chemin des capelles, Toulouse F-31076, France; 3 Centre de Microscopie Electronique Appliquée à la Biologie, Faculté de Médecine de Rangueil, 133 route de Narbonne, Toulouse, F-31062, France

## Abstract

Myxoma virus (MYXV), a leporide-specific poxvirus, represents an attractive candidate for the generation of safe, non-replicative vaccine vector for non-host species. However, there is very little information concerning infection of non-laboratory animals species cells with MYXV. In this study, we investigated interactions between bovine cells and respectively a wild type strain (T1) and a vaccinal strain (SG33) of MYXV. We showed that bovine KOP-R, BT and MDBK cell lines do not support MYXV production. Electron microscopy observations of BT-infected cells revealed the low efficiency of viral entry and the production of defective virions. In addition, infection of bovine peripheral blood mononuclear cells (PBMC) occurred at a very low level, even following non-specific activation, and was always abortive. We did not observe significant differences between the wild type strain and the vaccinal strain of MYXV, indicating that SG33 could be used for new bovine vaccination strategies.

## Background

Until now, most of the ruminant vaccines use attenuated strains of pathogens, and for that reason, naturally infected and vaccinated animals cannot easily be differentiated. Development of recombinant vaccines for ruminant species would help to implement vaccine policies. The development of poxviruses as vectors for producing recombinant vaccines is well documented [1-6]. Although vaccinia virus was the first and most extensively developed poxvirus vector, concerns over its use in immunocompromised persons and its broad host-range specificity [7] had led to search for alternative poxviruses which might prove more suitable vectors. Myxoma virus (MYXV), a leporipoxvirus causing myxomatosis, a highly lethal disease of European rabbit, could be an interesting tool for animals vectored vaccination. MYXV attenuated strains were shown to be efficient vaccine vector to vaccinate its natural host against both myxomatosis and rabbit viral hemorrhagic disease [8,9]. Recently, MYXV was successfully developed as a non replicative vector to vaccinate cats against feline calicivirus [10,11]. However, for each target species, evaluation of host restriction is of importance for the development of safe and potent vaccine vectors. MYXV is reported to be restricted to rabbits *in vivo *[12] and to replicate *in vitro *in some non natural host cell lines such as simian BGMK and some cancer cells [13]. No information concerning interactions between MYXV and bovine cells is available yet. In this study, we characterized the infection of bovine cell lines and bovine peripheral blood mononuclear cells (PBMC) with MYXV. By comparing two different MYXV strains (a wild-type strain (T1) and a cell-cultured attenuated vaccinal strain (SG33) [14]) we verified the stability of the viral tropism *in vitro*.

## Findings

**Figure 1:**
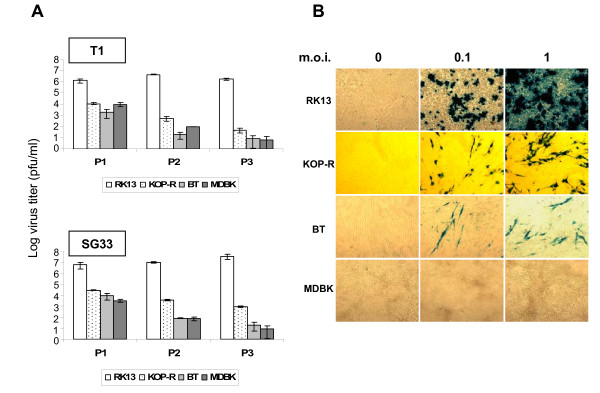
**Permissivity of bovine cell lines for myxoma virus**. Bovine cells were maintained in DMEM (KOP-R and BT) or MEM (MDBK) supplemented with 10 % foetal calf serum (FCS). **A. **Virus production in different bovine cell lines over three passages. Cells were first inoculated with either T1 (top) or SG33 (bottom) MYXV strain at a m.o.i. of 1, washed, cultured in DMEM, 5 % FCS for 3 days and frozen (P1). In subsequent infections, 1/5 of the material from the previous frozen culture was used for infection (P2 and P3). Titers were determined by serial dilution-titration on RK13 cells. The values correspond to a mean of at least two independent experiments. Error bars correspond to the standard error of the mean. **B. **Rabbit and bovine cells were infected with the T1-TK::LacZ (m.o.i. of 0.1 and 1). Twenty-four hours p.i., they were fixed with 2.5 % glutaraldehyde for 15 minutes at room temperature and stained with 2 mg/ml X-Gal in 2 mM MgCl_2_, 5 mM K_4_Fe(CN)_6_.3H_2_O, 5 mM K_3_Fe(CN)_6 _in PBS for 4–10 hours and observed by microscopy. Microscope: Leica; Magnification: 100.

Three bovine cell lines were tested for MYXV permissivity: KOP-R cells (RIE 244, CCLV Federal Research Centre for Virus Diseases of Animals, Island Riems), BT cells (ATCC CRL-1390) and MDBK cells (ATCC CCL-22). Each cell line was infected at a multiplicity of infection (m.o.i.) of 1 and cultured for 72 h. Then, infected cells were lysed by three freeze/thaw cycles. One fifth of each cell lysate was inoculated to new cells and further incubated for 72 h. Virus productions were determined by serial dilution-titration of each cell lysate on permissive rabbit RK13 cells (ATCC CCL-37) (Figure 1A).

In RK13 cells, used as positive control, we observed a high virus titer maintained over the three passages for both MYXV strains (Figure 1A). In contrast, in the bovine cell lines, viral titers decreased during the three passages for both T1 and SG33 (Figure 1A). After three passages we measured a low virus titer for KOP-R, BT and MDBK indicating that both strains are not able to spread over serial passages.

To confirm these results, we infected the bovine cell lines or RK13 cells with T1 or SG33 recombinant viruses expressing the LacZ reporter gene driven by the late poxviral P11 promoter. Figure 1B shows the results obtained with the T1 recombinant virus. In bovine KOP-R and BT cell lines, at m.o.i. 0.1 or 1, only sparse infected cells were observed (β-galactosidase positive cells), whereas in MDBK cells, no β-galactosidase labelled cell was present, indicating no expression of late viral protein. However, early viral proteins could be detected (data not shown). Similar results were observed using SG33 (data not shown).

**Figure 2:**
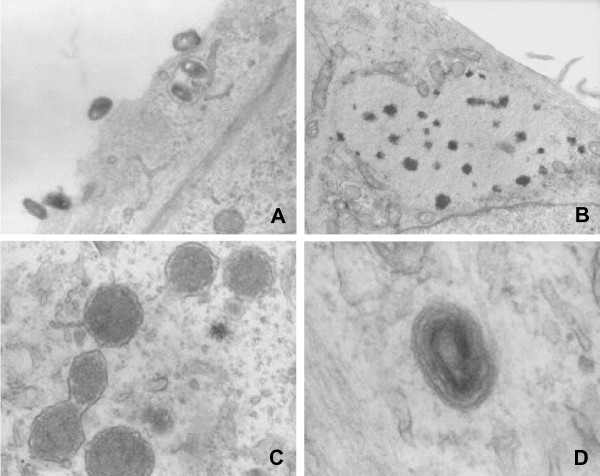
**Electron microscopy observations of MYXV BT infected cells. A**. Numerous virions are still adsorbed on the cell surface 8 h p.i.. **B. **Large cytoplasmic area without organite containing dense particles (5 h p.i.). **C. **Atypical Immature Virion (IV) (12 h p.i.). **D. **Intracellular Enveloped Virion (IEV) (12 h p.i.). Microscope: Hitachi UI12A, Magnifications, **A**: 35000; **B**: 15000; **C **and **D**: 90000.

We next performed an electron microscopy study in MYXV infected BT cells (Figure 2). BT cell monolayers were infected with T1 at a m.o.i. of 8. Five, 8, 12 and 24 hours following infection, cells were fixed and processed for electron microscopy as previously described [15]. We observed a lot of virions adsorbed on the cell surface, throughout the kinetic (Figure 2A, and not shown). Viral penetration appeared to be less efficient than with permissive cells [15]. Five hours post-infection (p.i.), we could observe very rare uncoating figures in the cytoplasm and large areas free from organites, containing electron-dense particles (Figure 2B). The first immature virions (IV) could be observed from 12 h p.i. only, 4 hours later than in permissive cells [15]. Most of these IV had an atypical electron-dense aspect characterized by an irregular and not well-defined membrane (Figure 2C). Intracellular enveloped virions (IEV) (Figure 2D) could rarely be observed and no intracellular mature virion (IMV) was detected. Enveloped mature virions (CEV, EEV) release was not detected. These results suggest that in addition to poor penetration efficiency, the late steps of viral maturation are impaired in BT cells.

To evaluate MYXV infection in blood primary bovine cells, peripheral blood mononuclear cells (PBMC) were infected. Bovine blood was collected in EDTA tubes, diluted (1:2), loaded on a density gradient (FicollPaque Plus, Amersham) and centrifugated at 900 g for 20 minutes. PBMC were then harvested, washed in PBS, recovered by centrifugation at 870 g for 10 minutes and cultured. To detect infected cells by flow cytometry, we used a recombinant SG33 virus expressing the enhanced green fluorescent protein (GFP) under the control of strong early/late vaccinia virus P7.5 promoter. The GFP encoded gene was inserted into the M11L/MGF locus. We also used the T1-Serp2-GFP recombinant virus which expresses the fused protein Serp2-GFP.

**Figure 3:**
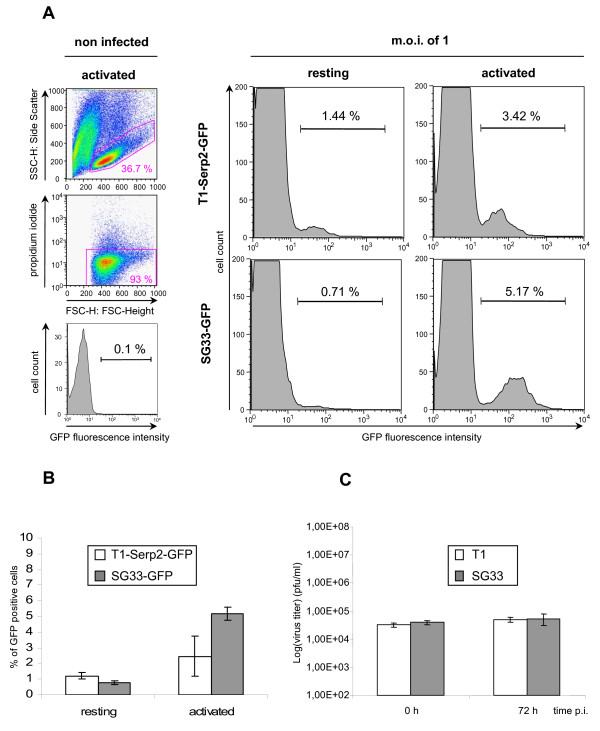
**Infection of bovine PBMC**. PBMC were isolated from bovine (Holstein Breed) whole blood on Ficoll density gradient and cultured with RPMIc containing RPMI 1640 with Glutamax, 25 mM Hepes (Gibco-BRL) supplemented with 10 % FCS, 1 % sodium pyruvate, 1 % non essential amino acids, 1 % β-mercapto-ethanol, 100 units/ml penicillin, 100 μg/ml streptomycin. When indicated, cells were activated 4 h before infection, using 125 ng/ml of phorbol myristate acetate and 50 ng/ml of ionomycine. To determine cell viability, propidium iodide (BD Biosciences Pharmingen) was added at 1 μg/ml just before acquisition. Acquisition was performed using a FACScalibur (Becton Dickinson). Dead cells and debris were excluded by appropriate gating and 30000 events were collected. Analysis was performed using CellQuestPro and Flowjo Software. **A. **Cells were infected at m.o.i. of 1 (T1-Serp2-GFP or SG33-GFP) and collected 16 h p.i.. Results shown are representative of three experiments. **B**. Cells were infected at m.o.i. of 1 (T1-Serp2-GFP or SG33-GFP), collected 16 h p.i and analyzed for GFP expression by flow cytometry. The percentages indicated represent an average of at least 3 independent experiments. Errors bars correspond to the standard error of the mean. **C. **PBMC were inoculated with T1 or SG33 (m.o.i. = 1), adsorption occurring 90 min at 4°C. Cells were washed twice and cultured. Virus productions at 0 h or 72 h post-infection were determined by serial dilution-titration on RK13 cells. The experiment was repeated three times. Error bars correspond to the standard error of the mean.

Resting PBMC were infected with T1-Serp2-GFP or SG33-GFP at a m.o.i. of 1 (Figure 3A). Cells were collected 16 h p.i., and infection levels were determined by counting living GFP-positive cells (Figure 3A). We observed that only a small fraction of bovine PBMC was susceptible to MYXV infection. An average of 1.2 % and 0.8 % of GFP-positive resting cells was detected for T1-Serp2-GFP and SG33-GFP respectively (Figure 3B). As activation may be required to allow infection by poxviruses [16], chemically-activated bovine PBMC were also infected at the same m.o.i.. The percentage of GFP-positive cells remained low following activation, as only 2.4 % and 5.1 % for T1-Serp2-GFP and SG33-GFP of positive cells were detected respectively (Figure 3B). The infection level remained below 5 % with an m.o.i. up to 10 (data not shown). In contrast to the infection level in activated rabbit PBMC (about 50 % of infected cells) (data not shown), activation have very low effect on bovine leukocytes infection with MYXV.

In activated bovine PBMC, T1 or SG33 production was analyzed by infection at a m.o.i. of 1, and virus titration on RK13 cells (Figure 3C). No significant increase of viral titers between 0 h and 72 h p.i. was noticed indicating that activated bovine PBMC are not permissive to MYXV infection.

## Conclusion

In this study, we investigated the interactions between bovine cells (cell lines and PBMC) and MYXV wild type (T1) strain or vaccinal (SG33) strain. In bovine cell lines, serial viral passages analysis and infection with both T1 and SG33 expressing LacZ gene showed that these cells failed to support spread of either MYXV strain. Electron microscopy study of BT-infected cells enabled us to identify at least two blocking events, the first one involving virus entry. Indeed, we observed many viral particles adsorbed on the cell surface throughout the experiment but very few infected cells. This result indicates that MYXV can bind to the cell surface, but enters the cells with low efficiency. The second blocking event involves the final steps of virus maturation, as numerous electron dense particles, similar to those already described in non-permissive cells infected with MVA [17-20] were present. In addition, very few IEV particles and no mature virions could be observed. As already suggested, these dense particles are more likely the products of defective virions morphogenesis [20]. The very low level and abortive infection of bovine PBMC make it impossible for MYXV to disseminate via leukocytes in these animal species. Taken together, these results are compatible with the potential use of the SG33 MYXV strain as a safe non replicative vector for bovine vaccination.

## Competing interests

The author(s) declare that they have no competing interests.

## Authors' contributions

BP conducted all the experiments, except electron microscopy analyses. JLD, AA and JG performed electron microscopy studies. GF contributed to PBMC infection studies. GF and SB coordinated the research. BP and SB wrote the manuscript.

All authors read and approved the final manuscript.
